# Complete Genome Sequence of an *Acinetobacter* Strain Harboring the NDM-1 Gene

**DOI:** 10.1128/genomeA.00023-12

**Published:** 2013-04-18

**Authors:** Yang Sun, Yang Song, Hongbin Song, Jun Liu, Pengzhi Wang, Shaofu Qiu, Shuo Chen, Lingwei Zhu, Xue Ji, Zhongqiang Wang, Nan Liu, Liliang Xia, Weijun Chen, Shuzhang Feng

**Affiliations:** Institute of Military Veterinary, AMMS, Key Laboratory of Jilin Province for Zoonosis Prevention and Control, Changchun, Chinaa;; Beijing Institute of Genomics, Chinese Academy of Sciences, Beijing, Chinab;; Institute of Disease Prevention and Control, Academy of Military Medical Sciences, Beijing, Chinac

## Abstract

The NDM-1 gene is a significant public health concern. *Acinetobacter* is one of the most prevalent opportunistic pathogens causing recent nosocomial infections with NDM-1, and drug-resistant strains pose serious threats to public health worldwide. Herein, we present the genomic sequence of *Acinetobacter calcoaceticus* subsp. *anitratus* XM1570, a multidrug-resistant isolate that carries the *bla*_NDM-1_ gene.

## GENOME ANNOUNCEMENT

The NDM-1 gene (6) is present worldwide in diverse pathogens, including *Acinetobacter baumannii, Enterobacter cloacae*, *Escherichia coli*, *Klebsiella pneumoniae*, and *Pseudomonas aeruginosa* (2–5). Bacteria carrying the *bla*_NDM-1_ gene have been termed “superbugs” and have attracted worldwide research attention. The gene is usually present on a large plasmid (1, 3, 5).

*Acinetobacter calcoaceticus* subsp. *anitratus* XM1570 was cultivated from a sputum sample of a lung cancer patient treated in a hospital in Xiamen, Fujian province, China, in 2010. The male patient was initially admitted because of cold symptoms. The patient was treated with clarithromycin, imipenem, and cilastatin sodium via injection, and he was then diagnosed with lung cancer, right pleural metastasis, and pneumonia. A sputum culture yielded a multidrug-resistant *Acinetobacter* strain that was resistant to beta-lactam antibiotics and carbapenems. The genome of this isolate, which harbors the *bla*_NDM-1_ gene, was completely sequenced. The total length of the assembled genome is 4,100,402 bp, and 3,959 coding sequences (CDSs) were identified. Further, a 47,274-bp plasmid carrying *bla*_NDM-1_ was identified. Our sequence data will be more valuable when further studies on the horizontal transfer of *bla*_NDM-1_ are conducted.

Our strain is very similar to *Acinetobacter calcoaceticus* PHEA-2. This drug-resistant strain, which has been identified in many Chinese hospitals, belongs to the *A. calcoaceticus-A. baumannii* (ACB) complex (7). This group includes *Acinetobacter* genomic species 3, *A. baumannii*, *Acinetobacter* genomic species 13TU, and *A. calcoaceticus*; it is difficult to phenotypically distinguish between these species (4). As is true of *A. baumannii, A. calcoaceticus* is a causative agent of both community-acquired and nosocomial infections (2). Intensive phylogenetic analysis has recently suggested that strain PHEA-2 is a novel species (termed *A. calcoaceticus*). Herein, we present the draft genomic sequence of the *bla*_NDM-1_-bearing strain *Acinetobacter calcoaceticus* subsp. *anitratus* XM1570.

Whole-genome sequencing was performed using the Illumina HiSeq 2000 method. Ultimately, 410 Mb and 210 Mb of high-quality data were generated from two libraries; the read lengths were both 90 bp. The paired-end reads were first assembled *de novo* using SOAP*denovo* v1.05. *Acinetobacter oleivorans* DR1 and contigs were next manually connected with reference to paired-end relationships at the 2-kb level. After all sequence data were evaluated, we identified putative protein-encoding sequences using Glimmer 3.0 (3), and then used BLAST to identify homologies with publically available database data prior to functional annotation. rRNAs and tRNAs were identified using RNAmmer 1.2 and tRNAscan-SE 1.3.1, respectively; ultimately, 63 tRNAs and three rRNAs were found.

Finally, we obtained 95 contigs with a total length of 4,076,308 bp, including one 47.3-kb circular contig identified as a *bla*_NDM-1_-bearing plasmid. A total of 3,959 CDSs were identified on the contigs. The *bla*_NDM-1_-bearing plasmid differed from that reported previously in *E. coli* (5). The *bla*_NDM-1_ region in the plasmid is located in an island of medium G+C content (40.83%); the average G+C percentage of the strain is 38.82%. β-lactamase and 16S rRNA methylase genes were sought using PCR and specific sequencing protocols to check whether the sequencing data are accurate.

### Nucleotide sequence accession numbers. 

The genome sequence of *Acinetobacter calcoaceticus* subsp. *anitratus* XM1570 has been deposited at DDBJ/EMBL/GenBank under the accession no. AMXH00000000. The version described in this paper is the first version, accession no. AMXH01000000.

## References

[1] Bonnin RA Poirel L Carattoli A Nordmann P 2012 Characterization of an IncFII plasmid encoding NDM-1 from *Escherichia coli* ST131. PLoS One 7:e34752 22511964 10.1371/journal.pone.0034752 PMC3325265

[2] Chen Z Qlu S Wang Y Wang Y Liu S Wang Z Du X Wang L Guo J Wang Z Liu N Yuan J Song H Huang L 2011 Coexistence of *bla*_NDM-1_ with the prevalent *bla*_OXA23_ and *bla*_IMP_ in pan-drug resistant *Acinetobacter baumannii* isolates in China. Clin. Infect. Dis. 52:692–693 21292674 10.1093/cid/ciq231

[3] Ho PL Lo WU Yeung MK Lin CH Chow KH Ang I Tong AH Bao JY Lok S Lo JY 2011 Complete sequencing of pNDM-HK encoding NDM-1 carbapenemase from a multidrug-resistant *Escherichia coli* strain isolated in Hong Kong. PLoS One 6:e17989 21445317 10.1371/journal.pone.0017989 PMC3061923

[4] Jovcic B Lepsanovic Z Suljagic V Rackov G Begovic J Topisirovic L Kojic M 2011 Emergence of NDM-1 metallo-β-lactamase in *Pseudomonas aeruginosa* clinical isolates from Serbia. Antimicrob. Agents Chemother. 55:3929–3931 21646490 10.1128/AAC.00226-11 PMC3147624

[5] Kumarasamy KK Toleman MA Walsh TR Bagaria J Butt F Balakrishnan R Chaudhary U Doumith M Giske CG Irfan S Krishnan P Kumar AV Maharjan S Mushtaq S Noorie T Paterson DL Pearson A Perry C Pike R Rao B Ray U Sarma JB Sharma M Sheridan E Thirunarayan MA Turton J Upadhyay S Warner M Welfare W Livermore DM Woodford N 2010 Emergence of a new antibiotic resistance mechanism in India, Pakistan, and the UK a molecular, biological, and epidemiological study. Lancet Infect. Dis. 10:597–602 20705517 10.1016/S1473-3099(10)70143-2 PMC2933358

[6] Marra A 2011 NDM-1: a local clone emerges with worldwide aspirations. Future Microbiol. 6:137–141 21366414 10.2217/fmb.10.171

[7] Pfeifer Y Wilharm G Zander E Wichelhaus TA Göttig S Hunfeld KP Seifert H Witte W Higgins PG 2011 Molecular characterization of *bla*_NDM-1_ in an *Acinetobacter baumannii* strain isolated in Germany in 2007. J. Antimicrob. Chemother. 66:1998–2001 21693460 10.1093/jac/dkr256

